# Biological Toxicity and Inflammatory Response of Semi-Single-Walled Carbon Nanotubes

**DOI:** 10.1371/journal.pone.0025892

**Published:** 2011-10-07

**Authors:** Eun-Jung Park, Jinkyu Roh, Soo Nam Kim, Min-Sung Kang, Byoung-Seok Lee, Younghun Kim, Sangdun Choi

**Affiliations:** 1 Department of Molecular Science and Technology, Ajou University, Suwon, Korea; 2 Department of Chemical Engineering, Kwangwoon University, Seoul, Korea; 3 Inhalation Toxicology Center, Korea Institute of Toxicology, Jeongeup, Korea; RMIT University, Australia

## Abstract

The toxicological studies on carbon nanotubes (CNTs) have been urgently needed from the emerging diverse applications of CNTs. Physicochemical properties such as shape, diameter, conductance, surface charge and surface chemistry of CNTs gained during manufacturing processes play a key role in the toxicity. In this study, we separated the semi-conductive components of SWCNTs (semi-SWCNTs) and evaluated the toxicity on days 1, 7, 14 and 28 after intratracheal instillation in order to determine the role of conductance. Exposure to semi-SWCNTs significantly increased the growth of mice and significantly decreased the relative ratio of brain weight to body weight. Recruitment of monocytes into the bloodstream increased in a time-dependent manner, and significant hematological changes were observed 28 days after exposure. In the bronchoalveolar lavage (BAL) fluid, secretion of Th2-type cytokines, particularly IL-10, was more predominant than Th1-type cytokines, and expression of regulated on activation normal T cell expressed and secreted (RANTES), p53, transforming growth factor (TGF)-β, and inducible nitric oxide synthase (iNOS) increased in a time-dependent manner. Fibrotic histopathological changes peaked on day 7 and decreased 14 days after exposure. Expression of cyclooxygenase-2 (COX-2), mesothelin, and phosphorylated signal transducer and activator of transcription 3 (pSTAT3) also peaked on day 7, while that of TGF-β peaked on days 7 and 14. Secretion of histamine in BAL fluid decreased in a time-dependent manner. Consequently, we suggest that the brain is the target organ of semi-SWCNTs brought into the lung, and conductance as well as length may be critical factors affecting the intensity and duration of the inflammatory response following SWCNT exposure.

## Introduction

Because of advances in scientific research and technology, nanomaterials, including carbon nanotubes (CNTs), are now used in biomedical, cosmetic, aeronautics, electronics, and other industries. Further, their effects on human health are also gaining enormous attention from the public.

Single-walled carbon nanotubes (SWCNTs) have been included in the priority list of OECD (Organization for Economic Co-operation and Development) and have been introduced in many new technologies for various industrial applications. Many epidemiological and toxicological studies have reported on the adverse health effects of SWCNTs [1], [2], [3], [4]. According to these reports, CNTs cause adverse health effects such as pulmonary inflammation, granulomatous lesions, fibrosis, and mesothelioma by inducing oxidative stress and free radical generation after inflow of SWCNTs into the lung [2], [5], [6], [7], [8]. When experimental animals were treated with SWCNTs, crocidolite asbestos, and ultrafine carbon black of the same concentration, SWCNTs caused the greatest histopathological changes, and lung tissue proteins affected by SWCNT exposure largely represent cellular processes affected by asbestos as well [9]. Nitric oxide (NO) production increased and mitochondrial activity decreased after an engineered human lung was exposed to SWCNTs [10], and treatment with SWCNTs and ovalbumin (OVA) strongly promoted an OVA-specific allergic response [11]. The results of microarray analysis following intratracheal instillation of SWCNTs suggest that an immunotoxicological mechanism may explain the chronic pulmonary inflammation and granuloma formation caused by SWCNTs *in vivo* [1]. Pharyngeal aspiration of SWCNTs also elicited unusual pulmonary effects in mice, combining a robust but acute inflammation with early onset yet progressive fibrosis and granulomas [6]. In addition, a single intrapharyngeal instillation of SWCNTs induced activation of heme oxygenase-1 (HO-1) in HO-1 reporter transgenic mice and aortic mtDNA damage in C57BL/6 mice [2].

The physicochemical properties of manufactured nanomaterials can vary according to manufacturing methods, and although the raw materials are the same, the toxicity can vary as well. Therefore, physical properties such as surface chemistry, conductance, diameter, structure, and shape together with the components of raw materials are considered important factors that depend on toxicity. SWCNTs are applied for the production of diverse products and the semiconductor industry is one of the main fields that use SWCNTs. In a previous study using pristine SWCNTs, which were a mixture of conductive (or metallic) and semi-conductive (or semi-metallic) components, we demonstrated that SWCNTs instilled intratracheally induced early lung fibrosis and chronic tissue damage [12]. Since semi-SWCNTs have less available charge density at the Fermi level than metallic-SWCNTs, they have very different chemical reactivity [13], [14], [15]. In the current study, we separated only semi-conductive components from the SWCNT mixture containing conductive and semi-conductive components in order to focus on the role of conductance in SWCNT toxicity. We then instilled a single intratracheal dose into mice at a concentration of 100 µg/kg and killed the mice on days 1, 7, 14 and 28 to investigate immunological and general toxicological changes with time. Body weight, organ weight, hematological changes, cytokine levels in bronchoalveolar lavage (BAL) fluid and blood, histopathological changes, and protein expression in lung tissue were determined at each time point.

## Materials and Methods

### Animals and housing conditions

Five-week-old ICR mice were purchased from Orient Bio Inc. (Gyeonggi-do, Korea) and acclimated to room conditions for 2 weeks prior to the initiation of the study. The environmental conditions were controlled at a constant temperature of 23±3°C, relative humidity of 55±10%, and a light/dark cycle of 12 h with 150–300 Lux, and ventilation 10–20 times/h. Three animals per cage were housed in suspended, stainless-steel wire cages (W×L×H = 255×465×200 mm) during the acclimation, pre-treatment, and treatment periods. Each animal was identified by a tail tattoo and a cage card. Gamma-ray-irradiated standard laboratory rodent pellet diet (PMI Nutrition International, Richmond, IN, USA) and municipal tap water sterilized with ultraviolet light were provided to the animals *ad libitum*. All experiments were performed in accordance with the guidelines and regulations of the Korea Institute of Toxicology and approved by the Institutional Animal Care and Use Committee (IACUC) (Approval Number: BJ09051). All animal facilities in this study were accredited by the Association for Assessment and Accreditation of Laboratory Animal Care International (AAALAC).

### Preparation of test material

SWCNTs (ASP-100F) were obtained from Hanhwa Nanotech Korea. Their metal content was approximately 10% of their weight, and they were 1.2 nm in diameter and 2–10 µm in length. Semi-SWCNTs were selectively separated from the mixture of metallic and semi-metallic SWCNTs using Yang's method [16]. Briefly, SWCNTs were treated with a mixture of HNO_3_ (Sigma Aldrich Korea) and H_2_SO_4_ (Sigma Aldrich Korea) (1∶9) with stirring for 12 h, followed by filtration using a 10-µm membrane filter and then washed several times with deionized water. SWCNTs on the membrane filter (semi-SWCNTS) were re-dispersed into 2 wt% sodium dodecyl sulfate (SDS; Sigma Aldrich Korea) solution by sonication (ULH-700S; ULSSO HI-Tech, Korea) for 30 min at 4 kHz (20% PWR) because SWCNTs are extremely hydrophobic and immiscible in water. Then, the solution was diluted with 2× phosphate-buffered saline (PBS). To confirm the conductive state of the SWCNTs, Raman peak analysis was carried out with Raman spectroscopy (B, T64000; HORIABA Jobin Yvon, France). The size of the SWCNTs was analyzed using transmission electron microscopy (JEM1010; JEOL, Japan) and dynamic light scattering (ELS-8000; Otsuka Electronics, Japan).

### Intratracheal instillation

To observe time-dependent changes, 15 mice were treated per time point. Intratracheal instillation was performed by a special technician from the Korea Institute of Toxicology, one of the GLP (Good Laboratory Practice) institutes in Korea, and semi-SWCNTs were delivered using a 24-gauge catheter at a 100 µg/kg dose for intratracheal administration under light tiletamine anesthesia. The animals were killed on days 1, 7, 14 and 28 after exposure. The control group (3 mice per time point) was treated with a vehicle control solution that was manufactured by the same method.

### Measurements of body weight and organ weight

Body weights were measured prior to necropsy on days 1, 7, 14 and 28. Absolute organ weights were measured, and relative organ weights (organ-to-body weight ratios) were calculated from the terminal body weight before necropsy of the livers, lungs, brain, thymus, heart, kidneys, spleen and testes. The paired organs were weighed together.

### Hematological examination

Blood samples (approximately 0.5 mL) were collected from the caudal vena cava of each animal and put into a blood-collecting tube containing ethylenediaminetetraacetic acid-2K for hematology. Samples were analyzed by ADVIA120 (Hematology System, Bayer, USA) in order to determine the white blood cell count (WBC), red blood cell count (RBC), hemoglobin concentration, hematocrit, mean corpuscular volume, mean corpuscular hemoglobin, mean corpuscular hemoglobin concentration, platelets, neutrophils, lymphocytes, monocytes, eosinophils, basophils, and large unstained cells.

### Collection and staining of BAL fluid cells

At the selected time intervals after administration, about 1.2 mL of blood was collected per mouse from the saphenous vein under isoflurane anesthesia. Whole blood was centrifuged at 3,000 rpm for 10 min to separate out serum, and 500–600 µL of serum was obtained from each mouse. The BAL fluid was obtained by cannulating the trachea and lavaging the lungs with 1 mL of cold sterile Ca^2+^/Mg^2+^-free PBS (0.15 M, pH 7.2). Approximately 500–600 µL of BAL fluid was harvested per mouse and then centrifuged at 3,000 rpm for 10 min. The resuspended pellets were centrifuged using Shandon Cytospin 4 (Thermo Scientific, USA) and stained with Wright-Giemsa.

The blood harvested from the 3 mice at each time-point and 1/2 the blood harvested from the control at each time point was used for hematological analysis (n = 3). The samples harvested from 3 exposed mice at each time point were pooled into 4 test samples for further analysis (n = 4), and the control samples were pooled by each time point (n = 4).

### Measurement of cytokines

The concentrations of each cytokine in the supernatant of the BAL fluid and serum were determined using commercially available enzyme-linked immunosorbent assay (ELISA) kits (eBioscience, San Diego, CA, USA). First, each well in the microplates was coated with 100 µL of capture antibody and incubated overnight at 4°C. After washing and blocking with assay diluent and BAL fluid, serum or standard antibody was added to the individual wells. The plates were then maintained at room temperature for 2 h. Next, the plates were washed, and biotin-conjugated detecting antibody was added to each well. Then, the plates were incubated at room temperature for 1 h. After incubation, the plates were washed again and further incubated with avidin–horseradish peroxidase for 30 min before detection with 3,3′,5,5′-tetramethylbenzidine solution. Finally, the reactions were stopped by adding 1 M H_3_PO_4_, and the absorbance at 450 nm was measured with an ELISA reader (Molecular Devices, Sunnyvale, CA, USA). The amount of cytokine was calculated from the linear portion of the generated standard curve [17], [18].

### Protein expression in tissue

Lung tissue was homogenized with a protein extraction solution (PRO-PREP^TM^, Cat. No. 17081, iNtRON Biotechnology, Kyunggi, Korea), and the lysates were centrifuged at 13,000 rpm for 10 min. The protein concentration was measured by the Bradford method (Bio-Rad Protein Assay, Bio-Rad Laboratories Inc., Hercules, CA), and equal amounts of protein (40 µg) were separated on a 1% SDS/ polyacrylamide gel and then transferred to a nitrocellulose membrane (Hybond ECL; Amersham Pharmacia Biotech Inc., Piscataway, NJ, USA). Blots were blocked for 2 h at room temperature with 5% (w/v) non-fat dried milk in Tris-buffered saline (10 mM Tris, pH 8.0, and 150 mM NaCl) solution containing 0.05% Tween-20. The membranes were immunoblotted with primary specific antibodies: rabbit polyclonal antibody for cyclooxygenase 2 (COX-2; 1∶500 dilution; Cayman Chemical, MI, USA), rabbit monoclonal antibody for β-actin (1∶2000 dilution; Cell Signaling Technology, Inc. Beverly, MA, USA), mouse monoclonal antibodies for phosphorylated signal transducer and activator of transcription 3 (pSTAT3; 1∶200 dilution; Santa Cruz Biotechnology Inc. CA, USA), mesothelin, matrix metalloproteinase (MMP)-9, p53 (1∶1000 dilution; Santa Cruz Biotechnology Inc.), inducible nitric oxide synthase (iNOS; 1∶1000 dilution; BD Biosciences, CA, USA), and regulated on activation normal T cell expressed and secreted (RANTES; 1∶4 dilution; eBioscience), and goat polyclonal antibody for transforming growth factor (TGF)- β1 (1∶1000 dilution; Santa Cruz Biotechnology Inc.). The blots were then incubated with the corresponding conjugated anti-mouse, anti-rabbit, or anti-goat immunoglobulin G-horseradish peroxidase (1∶2000 dilution; Santa Cruz Biotechnology Inc.). Immunoreactive proteins were detected with the ECL western blotting detection system.

### Histopathological examination

Histopathological analysis was performed at the Korea Institute of Toxicology (Daejeon, Korea) on tissue harvested from 6 mice per group. The lung, brain, and thymus from mice in the control group and the treated group were fixed with 10% neutral buffered formalin and processed using routine histological techniques. After paraffin embedding, 3-µm sections were cut and stained with hematoxylin and eosin (H&E) for histopathological evaluation.

### Statistical analysis

The results obtained from the chemically treated groups were compared to those of the control group. The values were compared using Dunnett's t-test after one-way analysis of variance (ANOVA), and levels of significance were represented compared to the control group.

## Results

### Physicochemical properties of semi-SWCNTs

**Figure 1:**
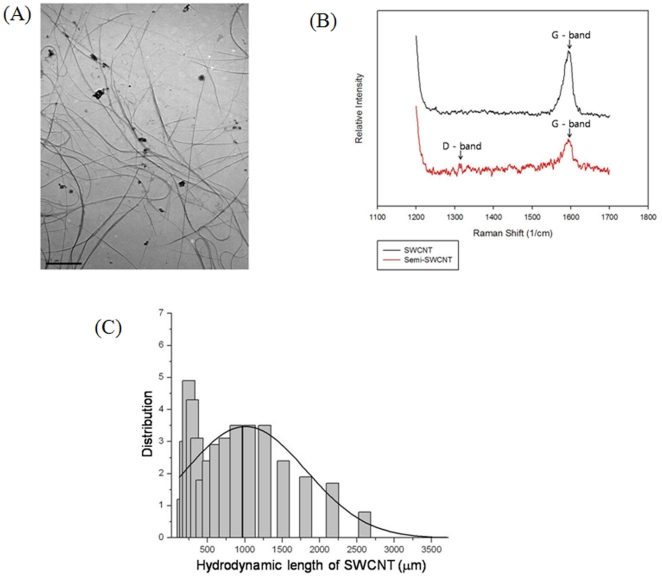
Physicochemical properties of semi-single-walled carbon nanotubes (semi-SWCNTs) suspended in phosphate-buffered saline (PBS). (A) Transmission electron microscopy (TEM) image, (B) Raman spectroscopy, and (C) Size distribution.

Figure 1 shows the following: (a) transmission electron microscopy image, (b) Raman spectra, and (c) average hydrodynamic size of SWCNTs. In Raman analysis, SWCNTs had an indigenous peak for the graphite-band at 1600 cm^–1^ because they have a regular carbon structure. However, the semi-SWCNT can exhibit an additional specific peak, disorder-band (1315 cm^-1^) peak, which is specific to the semi-structure. These results confirm that semi-SWCNTs were selectively separated. Semi-SWCNTs showed ca. 1.2 nm of diameter and 1 µm of hydrodynamic length and great stability in PBS with minute amounts of SDS (Fig. 1).

### Change of body weight caused by semi-SWCNT

**Figure 2:**
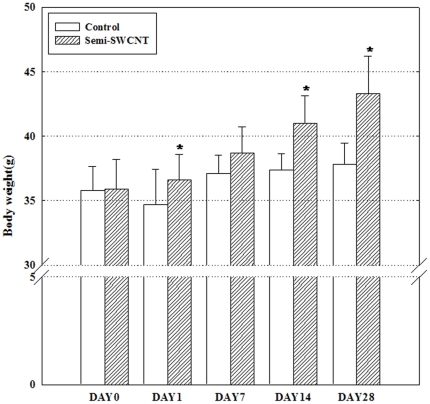
Changes in body weight following a single instillation of semi-SWCNTs. Mice (15/group) were a single intratracheal instilled with 100 µg/kg of semi-SWCNTs and then killed on the designated day (1, 7, 14, and 28). The control group (3 mice per time point) was treated with a vehicle control solution that was manufactured by the same method. *P<0.05.

Before exposure to semi-SWCNTs, body weights of the control and exposure groups were 35.8±1.9 g and 35.9±2.3 g, respectively (Fig. 2). The body weight of the mice in the control group increased about 2 g during the experimental period, whereas the body weight of mice in the exposure group rapidly increased to 36.6±1.96, 38.7±2.01, 41.0±2.13, and 43.3±2.91 g on days 1, 7, 14, and 28 after exposure, respectively.

### Change of relative organ weight

As shown in Table 1, the ratio of brain weight to body weight significantly decreased on days 1, 7 and 28; that of the thymus and heart significantly increased on days 1 and 14, respectively; and that of the kidney significantly decreased on days 1 and 7.

**Table 1:** Changes in relative organ weight following a single instillation of semi-single-walled carbon nanotubes (semi-SWCNTs).

		Brain	Lung	Thymus	Heart	Kidney	Spleen
DAY 1	C	1.371±0.129	0.665±0.211	0.132±0.016	0.454±0.041	1.524±0.237	0.293±0.044
	T	**1.269±0.039** *	0.642±0.090	**0.154±0.013** *	0.435±0.025	**1.441±0.007** *	0.307±0.050
DAY 7	C	1.357±0.103	0.654±0.092	0.110±0.056	0.471±0.033	1.639±0.233	0.372±0.115
	T	**1.307±0.053** *	0.584±0.114	0.132±0.059	0.459±0.050	**1.429±0.195** *	0.348±0.042
DAY 14	C	1.257±0.057	0.529±0.032	0.112±0.024	0.407±0.021	1.345±0.090	0.297±0.068
	T	1.230±0.103	0.534±0.052	0.125±0.034	**0.453±0.043** *	1.438±0.146	0.316±0.052
DAY 28	C	1.282±0.088	0.507±0.035	0.091±0.010	0.446±0.035	1.627±0.164	0.281±0.058
	T	**1.128±0.109** *	0.518±0.059	0.085±0.030	0.452±0.042	1.527±0.126	0.299±0.059

Note: Relative weight of each organ was computed using the formula (tissue weight/body weight) × 100. At each time point, the number in the exposure group was 15, and that in the control group was 3. C: Control group, T: semi-SWCNT group.

*P<0.05.

### Hematological changes

The number of WBCs began decreasing from day 7 after exposure, and a significant change was observed on day 28 (Table 2). Distribution of monocytes began increasing after exposure, and a significant change was observed on days 14 and 28. On day 28, the distribution of neutrophils and eosinophils significantly increased, whereas the distribution of lymphocytes significantly decreased.

**Table 2:** Hematological changes after a single instillation of semi-SWCNTs (n = 3, *; P<0.05).

		WBC	RBC	HGB	HCT	MCV	MCH	MCHC	PLT	RET	NEU	LYM	MON	EOS	BAS	LUC
		×10^3^ µL	×10^6^ µL	g/dL	%	fL	pg	g/dL	×10^3^ µL	%	%	%	%	%	%	%
DAY 1	C.	2.6±0.3	9.0±0.3	13.4±0.4	39.3±0.3	49.5±1.5	16.9±1.0	34.2±1.2	1423±242	3.3±0.5	21.9±8.8	71.6±8.3	1.2±0.4	4.3±0.2	0.4±0.2	0.5±0.3
	T	2.8±0.7	7.7±0.4	12.8±0.2	38.9±1.3	50.3±1.2	16.6±0.8	33.0±0.8	1391±145	3.3±0.3	19.8±6.0	74.2±6.5	1.9±1.0	2.8±0.7	0.2±0.1	1.0±0.6
DAY 7	C.	4.9±0.5	8.4±0.3	13.4±0.4	43.6±2.3	52.0±1.7	16.1±0.1	30.7±1.0	1518±21	5.6±1.3	57.4±2.3	38.8±2.8	1.0±0.2	2.3±0.6	0.1±0.1	0.4±0.4
	T	4.6±1.2	8.0±0.3	12.8±0.8	39.0±1.5	48.7±1.1	15.9±0.8	32.7±0.9*	1669±187	5.0±0.4	23.7±4.7*	71.0±4.0*	2.2±0.4	2.2±0.9	0.1±0.1	0.8±0.2
DAY 14	C.	4.5±1.2	8.3±0.7	13.3±1.4	40.6±4.3	49.1±1.5	16.1±0.3	32.8±1.0	1466±259	4.3±0.2	15.3±7.3	80.2±7.0	1.8±0.2	2.0±0.7	0.1±0.1	0.6±0.2
	T	4.0±1.1	8.9±0.3	14.3±0.3	45.2±3.2	50.6±2.4	16.1±0.2	31.8±1.5	1639±203	4.4±0.5	15.3±1.4	80.1±1.8	2.4±0.3*	1.5±0.4	0.1±0.1	0.5±0.2
DAY 28	C.	4.3±0.8	8.8±0.2	13.4±0.5	41.5±1.0	47.3±0.8	15.2±0.3	32.1±0.4	1442±30	3.1±0.2	15.5±7.1	80.8±7.0	2.0±0.7	1.1±0.2	0.0±0.1	0.5±0.2
	T	2.7±0.2*	8.3±0.5	13.1±0.3	41.8±0.3	50.4±2.5	15.8±0.8	31.4±0.9	1455±121	3.7±0.2	46.0±6.4*	46.1±6.5*	4.35±0.5*	2.8±0.4*	0.2±0.2	0.7±0.3

Note: C: control group; T: semi-SWCNT group; WBC: white blood cell; RBC: red blood cell; HGB: hemoglobin; HCT: hematocrit; MCV: mean corpuscular volume; MCH: mean corpuscular hemoglobin; MCHC: mean corpuscular hemoglobin concentration; PLT: platelets; RET: reticulocyte; NEU: neutrophils; LYM: lymphocytes; MON: monocytes; EOS: eosinophils; BAS: basophils; LUC: large unstained cells.

### Microscopic image of BAL fluid

**Figure 3:**
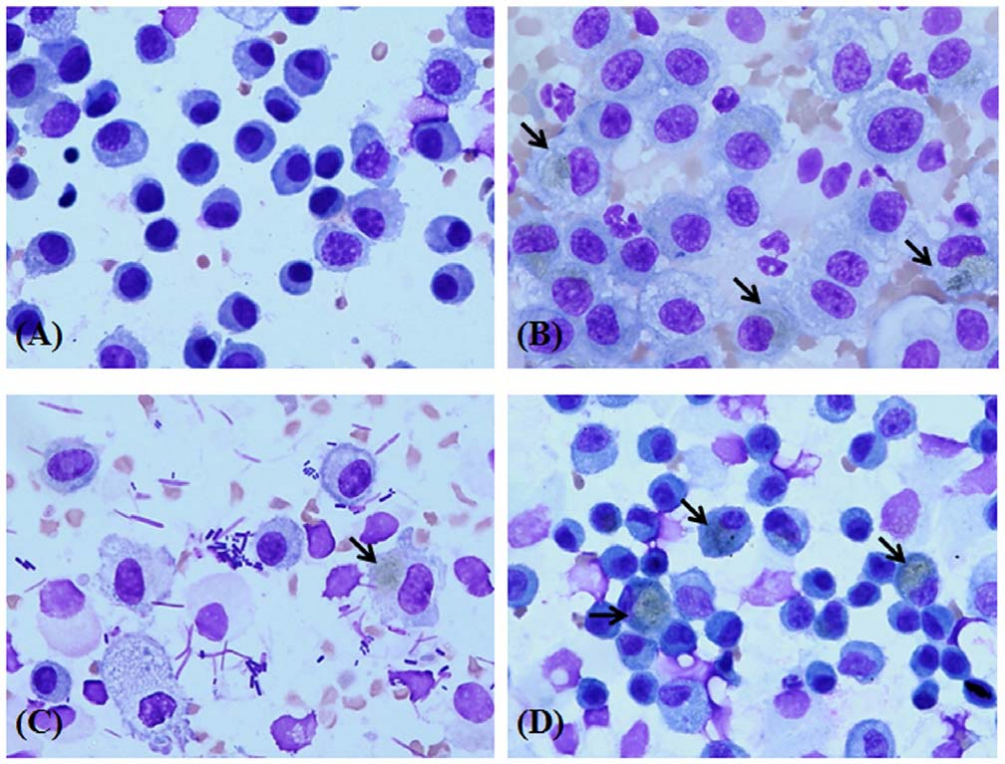
Images of cells in bronchoalveolar lavage (BAL) fluid following semi-SWCNT instillation. Mice were a single intratracheal instilled with 100 µg/kg of semi-SWCNTs, and the sample was harvested on the designated day (1, 7, 14, and 28). Results were confirmed for all mice, and representative images are shown. (A) solvent control, (B) day 1, (C) day 14, and (D) day 28.

BAL fluid harvested on day 1 generally contained a lot of blood, and objects that looked like pieces of broken sticks were found in BAL fluid on day 14 (Fig. 3). In addition, cells containing black lumps were observed during the entire experimental period.

### Cytokines in BAL fluid

The levels of all measured cytokines in the exposure group were significantly increased compared with those in the control during the entire experimental period. These levels peaked on day 14 after exposure (Table 3). However, the secretion of Th2-type cytokines was higher than that of Th1-type cytokines. On day 14, the concentrations of interleukin (IL)-1β, IL-6, and tumor necrosis factor (TNF)-α, which are pro-inflammatory cytokines, increased by 13.5-, 9.1-, and 9.6-fold, respectively, versus the control. The levels of IL-2, a Th0-type cytokine, in the exposure group was about 14.6-fold. The concentrations of IL-4, IL-5, IL-10, and IL-13, which are Th2-type cytokines, in the exposure group increased 12.3-, 8.6-, 14.5-, and 13.5-fold versus the control, respectively, whereas the concentrations of IL-12 and interferon-γ (IFN-γ), which are Th1-type cytokines, were about 1.9- and 6.8-fold, respectively, in the exposure group versus the control on day 14, respectively. The concentration of IL-17, a Th17-type cytokine, was about 6.3 fold in the exposure group versus the control.

**Table 3:** Changes in cytokine levels in bronchoalveolar lavage (BAL) fluid after a single treatment with semi-SWCNTs (n = 4).

	Control	DAY 1	DAY 7	DAY 14	DAY 28
IL-1	12.38±3.30	90.84±7.13	118.91±18.61	166.58±15.36	113.74±27.54
IL-6	33.13±11.38	284.71±27.11	298.85±32.55	300.57±26.63	300.57±66.46
TNF-α	4.57±2.21	24.16±3.01	31.51±2.20	43.91±5.55	25.65±4.70
IL-2	2.37±3.11	16.71±2.80	27.40±1.12	34.63±1.24	20.31±1.46
IL-12	16.84±2.28	23.58±1.88	25.26±0.21	31.58±2.58	27.15±3.98
IFN-γ	5.53±3.14	26.00±4.10	29.40±2.03	37.55±2.29	27.09±1.61
IL-4	6.41±1.20	52.14±1.35	70.04±0.55	78.97±4.46	62.99±2.60
IL-5	5.63±2.12	24.84±1.08	29.33±1.69	48.54±3.18	36.96±2.49
IL-10	104.98±14.76	998.37±121.56	975.23±166.96	1521.51±353.24	889.52±223.29
IL-13	1.60±0.01	12.04±0.39	16.59±1.01	21.65±0.55	14.84±1.10
IL-17	25.12±2.83	117.89±17.79	129.52±18.43	158.80±6.61	118.03±9.39

Note: BAL fluid was harvested at each time point and pooled (500 µL per mouse) to 4 test samples per group for further analysis. The level for each group was calculated in terms of mean±SD of the values measured. All treated groups except interleukin (IL)-12 had statistical significance of P<0.01 compared with the control group. TNF: tumor necrosis factor; IFN-γ: interferon-γ.

### Cytokines in the blood

As shown in Table 4, the concentration of IL-12 significantly increased during the entire experimental period, whereas marked levels of IL-6 were detected only on day 7 after exposure. Concentrations of IL-1, IL-6 (except day 7), TNF-α, and IL-17 were arithmetically significant, but low levels were detected.

**Table 4:** Changes in cytokine levels in the blood after a single treatment with semi-SWCNTs (n = 4).

	Control	DAY 1	DAY 7	DAY 14	DAY 28
IL-1	2.21±0.02	4.82±0.60**	4.11±0.85**	4.88±0.07**	3.35±0.01**
IL-6	ND	29.02±4.28**	3.71±0.07**	ND	2.70±0.04**
TNF-α	0.35±0.00	2.84±2.02	1.73±0.95*	4.02±0.09**	2.11±0.04**
IL-2	ND	ND	ND	ND	ND
IL-12	20.14±0.97	75.35±8.42**	43.88±3.85**	74.47±8.09**	68.00±9.74**
IFN-γ	ND	1.43±1.02	1.09±1.01	0.40±0.00	ND
IL-4	1.44±0.04	2.52±0.00	2.20±0.01	1.90±0.00	2.87±0.02
IL-5	ND	ND	ND	ND	ND
IL-10	ND	ND	ND	ND	ND
IL-13	ND	ND	ND	ND	ND
IL-17	2.74±0.00	6.56±1.07**	6.19±0.82**	14.09±2.36**	7.48±0.07**

Note: Serum was harvested at each time point and pooled (500 µL per mouse) to 4 test samples per group for further analysis. Level of each group was calculated as mean±SD of the values measured.

*P<0.05.

**P<0.01. IL: interleukin; TNF: tumor necrosis factor; IFN-γ: interferon-γ; ND: not defined.

### Secretion of TGF-β and histamine

**Figure 4:**
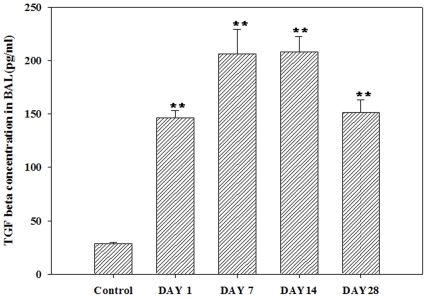
TGF-β levels in BAL fluid following semi-SWCNT instillation. BAL fluid and serum were obtained at each time point and pooled to get 4 samples per group for further analysis. TGF-β was not detected in blood at all time points. The level in each group is represented as mean±SD of the values measured. **P<0.01.

**Figure 5:**
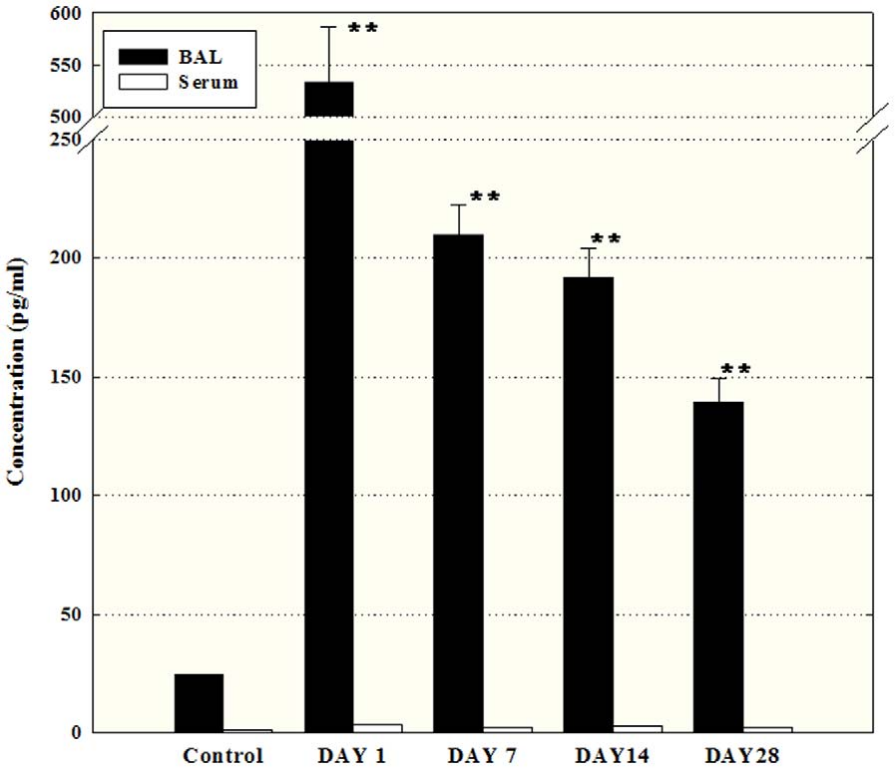
Histamine levels in BAL fluid and blood following semi-SWCNT instillation. BAL fluid and serum were harvested at each time point and pooled to get 4 samples per group for further analysis. The level in each group is presented as mean±SD of the values measured. *P<0.05.

The concentration of TGF-β in the BAL fluid of the exposed group was 146.3±6.6, 206.2±23.2, 208.3±14.3, and 151.6±11.3 pg/ml on days 1, 7, 14 and 28, respectively, whereas the concentration of TGF-β in the BAL fluid of the control group was 28.7±0.8 pg/ml (Fig. 4). In addition, the concentration of histamine in the BAL fluid decreased in a time-dependent manner compared with the level in the control (24.4±0.4 pg/ml) and was 532.3±54.1, 209.4±13.0, 191.5±12.4, and 138.9±10.0 pg/ml on days 1, 7, 14 and 28, respectively (Fig. 5). However, TGF-β and histamine were barely detected in the blood.

### Histopathology of lung tissue

**Figure 6:**
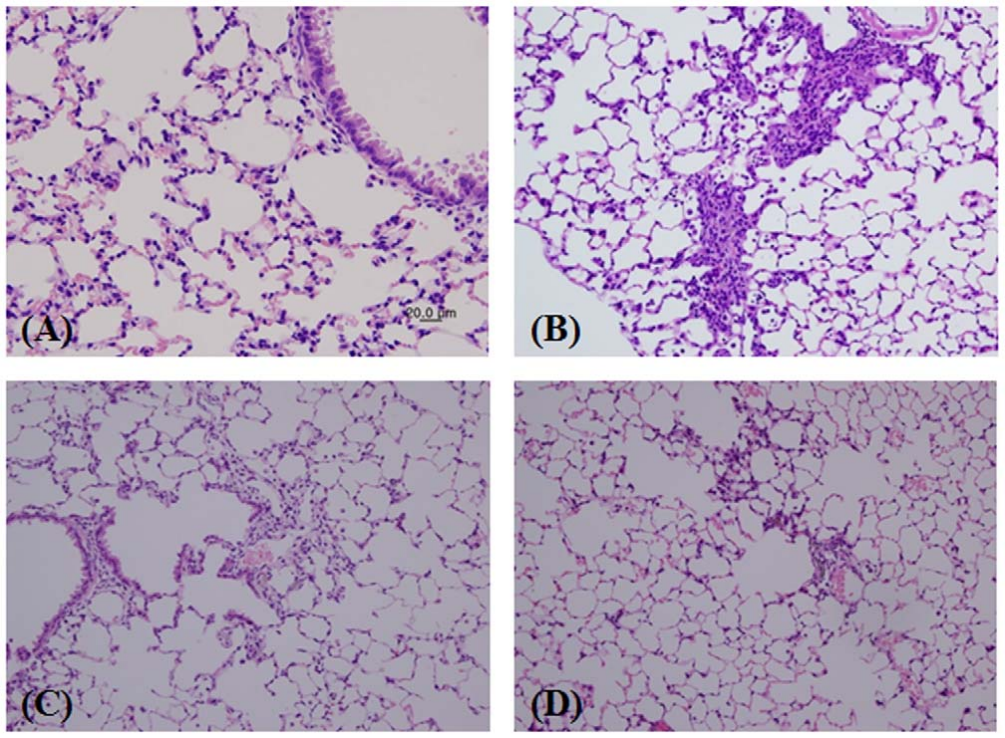
Histopathological changes in lung tissue after a single instillation of semi-SWCNTs. Lung sections were stained with hematoxylin and eosin stains (×200): (A) solvent control, (B) Day 7, (C) Day 14, and (D): Day 28. Results were confirmed for 6 mice in each group, and representative images are shown.

Pigmented macrophages were persistently observed from day 1 to day 28 after exposure (Fig. 6, Table 5). Pigmented macrophages were found around the alveolar region and alveolar wall on day 1 but were transferred to the alveolar wall and interstitial region as time progressed. Infiltration of inflammatory cells and fibrosis in the alveolar interstitial region was minimal to slight on day 7 after exposure and accompanied by infiltration of inflammatory cells containing alveolar macrophages and fibrosis surrounding pigmented macrophages. Histopathological changes on day 14 were similar to those on day 7, and infiltration of inflammatory cells and fibrosis were slight in only 1 mouse on day 28.

**Table 5:** Histopathological findings in lung tissues.

	Control		DAY 1		DAY 7		DAY 14		DAY 28	
	(a)	(b)	(a)	(b)	(a)	(b)	(a)	(b)	(a)	(b)
Pigmented macrophage			2/6	±	4/4	± or +	3/5	± or +	3/6	± or +
Inflammatory cell infiltration, Mixed					4/4	± or +	2/5	±	1/6	+
Fibrosis, Interstitial					3/4	± or +	1/5	+	1/6	±
Hemosiderin crystals, focal			1/6							

Note: a/b indicates (a) animals/histopathological sections were found positive amongst the total number of animals/histopathological sections, (b) evaluated.

### Protein expression following exposure to SWCNTs

**Figure 7:**
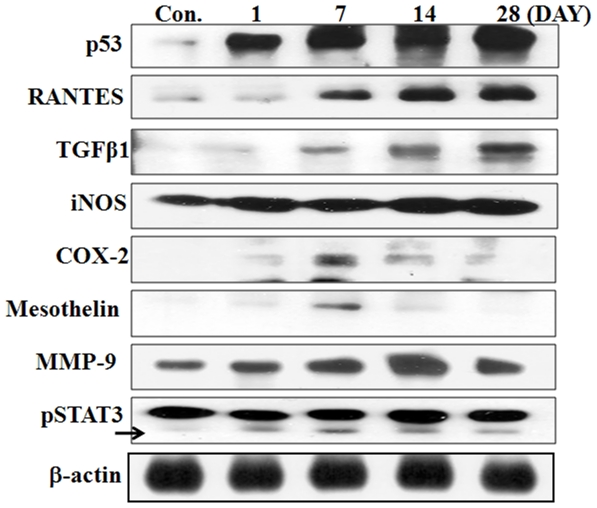
Changes in lung-tissue protein expression following a single instillation of semi-SWCNTs. Results were confirmed with multiple experiments and representative images are shown.

As shown in Fig 7, expression of p53, RANTES, TGF-β, and iNOS increased in a time-dependent manner, while that of COX-2, mesothelin, and pSTAT3 peaked on day 7, and that of MMP-9 peaked on day 14 after exposure.

## Discussion

The Globally Harmonized System of Classification and Labeling of Chemicals (GHS), which has been in full-scale enforcement since 2008, requires toxicity data for all components in the final products following a manufacturing process. In the most cases of existed chemicals, only chemical properties are changed by a manufacturing process, but both physical and chemical properties are changed for nanomaterials. These physical properties are known to be important factors that affect the toxicity of nanomaterials, including CNTs [3], [17], [19]. Therefore, the current GHS system has many limitations with regard to the classification of the toxicity of nanomaterials. In this study, we separated the semi-conductive component (semi-SWCNTs) from pristine SWCNTs using acid; semi-SWCNTs were longer than pristine SWCNTs [12]. The results of this study may reflect the roles played by length and conductance in SWCNT toxicity.

The prime target for CNT toxicity is believed to be the lung, where exposure may occur through inhalation, particularly in workplaces, because CNTs are very light. The physical nature of CNTs may also lead to penetration following deposition on an exposed site, inducing a chronic inflammatory response. Mercer et al. (2010) reported that 18%, 81.6%, and 0.6% of the MWCNT (multi-walled carbon nanotubes) lung burden was in the airway, the alveoli, and subpleural regions, respectively, on day 1 after exposure, and the density of infiltrates in the subpleural tissue and intrapleural space decreased by day 7 because of clearance by alveolar macrophages and/or lymphatics. However, the density of penetration increased to steady state levels in the subpleural tissue and intrapleural space from day 28 to day 56. MWCNTs of approximately 60 nm in diameter and 1.5 µm in median length deposited in the lungs of the rats were typically phagocytosed by the alveolar macrophages. These macrophages were subsequently accumulated in the alveoli until 6-month post-exposure [20]. In addition, aggregated SWCNTs induced granulomas that were mainly associated with hypertrophied epithelial cells, and dispersed SWCNTs induced diffuse interstitial fibrosis and alveolar wall thickening [6]. In this study, pigmented macrophages were observed around the alveolar region and alveolar wall on day 1 but were then found in the alveolar wall and interstitial area as time progressed. Furthermore, pigmented macrophages were continuously observed in BAL fluid from day 1 to day 28 after exposure, and fibrosis in the alveolar interstitial region was found from day 7 to day 28 after exposure. In addition, cell analysis of BAL fluid in a previous study, which examined pristine and acid-treated MWCNTs, revealed that pristine MWCNTs induce more severe acute inflammatory cell recruitment than acid-treated MWCNTs [19]. Hematological analysis in the current study revealed that the number of WBCs showed a time-dependent decrease relative to the control, whereas monocytes increased in a time-dependent manner relative to the control. In addition, significant hematological changes following semi-SWCNT exposure was mainly observed on day 28, whereas significant hematological changes following SWCNT exposure was mainly observed on day 7 (Supplementary Table S1). These results suggest that the inflammatory response to semi-SWCNTs may be extended relative to that to mixed SWCNTs.

Body weight and relative organ weight are important indices of toxicity. In our previous study, the group exposed to SWCNTs grew more rapidly compared with the control group, but the growth was not significant (Supplementary Fig. S1). The relative organ weight also was not consistently and significantly changed by SWCNT exposure (Supplementary Fig. S2). In this study, the body weight of mice significantly increased in the semi-SWCNT group compared with in the control group, and the relative weight of the brain significantly decreased in the semi-SWCNT group compared with in the control group. Furthermore, 2 mice exposed to semi-SWCNTs died on day 7 and 28, respectively. Acute inflammatory response was observed around the heart and thymus in the mouse that died on day 7, and inflammatory cell foci were observed in the kidney in the mouse that died on day 28. These results suggest that intratracheal instillation of semi-SWCNTs may induce a systemic inflammatory response, and the brain may be the target organ after respiratory exposure to semi-SWCNTs. Further study of the relationship between the increase in body weight and the decrease in relative weight of the brain is needed.

Cytokines are critical factors whose levels depend on the inflammatory response. In particular, the duration and pattern of cytokines secreted into BAL fluid play an important role in determining the course of pulmonary disease. In a previous study, exposure to SWCNTs resulted in early neutrophil accumulation (day 1), followed by lymphocyte (day 3) and macrophage (day 7) influx; these results were also accompanied by early elevation in the level of pro-inflammatory cytokines (day 1), followed by an increase in the level of fibrogenic TGF-β1 (peaked on day 7) [6]. In our previous study using mixed SWCNTs, pro-inflammatory cytokines rapidly increased on day 1 after instillation, and their levels were upregulated during the experimental period. IL-2 was maximum on day 7; IL-12 and IL-10 also rapidly increased on day 1 and remained at a similar level until day 28. IFN-γ and IL-4 were maximum on day 1, and IL-5 was maximum on day 7. IL-13 and IL-17 increased in a time-dependent manner [12]. In this study, semi-SWCNTs induced a significant increase in the levels of all cytokines measured in the BAL fluid during the experimental period; the levels of IL-2 and IL-10 were overwhelmingly upregulated in the exposure group compared with in the control group during the entire experimental period. In addition, expression of pSTAT3 and mesothelin protein began decreasing from day 7 after semi-SWCNT exposure, and time-dependent recruitment of monocytes into the bloodstream began occurring from day 1 after exposure. IL-10 is potent suppressor of macrophage function and is also known as cytokine synthesis inhibitor F. IL-10 is produced primarily by monocytes and to a lesser extent by Th2-type helper T cells, mast cells, and regulatory T cells and can inhibit synthesis of pro-inflammatory cytokines by cells such as macrophages and regulatory T cells. IL-10 also downregulates the expression of Th1-type cytokines and enhances B cell survival, proliferation, and antibody production. Furthermore, IL-10 can block nuclear factor-κB (NF-κB) activity and is involved in the regulation of the JAK(Janus kinase)–STAT signaling pathway [21], [22], [23], [24].

Histamine plays an important role in the pathogenesis of atopic asthma through differential regulation of T helper lymphocytes. Histamine enhances the secretion of Th2-type cytokines and inhibits the production of IL-2 and Th1-type cytokines [25], [26]. It has been shown that histamine can modulate the cytokine network through upregulation of PGE_2_ (prostaglandin E_2_) and NO [27]. In this study, secretion of histamine decreased in a time-dependent manner in the BAL fluid, despite predominance of Th2-type cytokines. This may be an effect of IL-2 and IFN-γ. Our hypothesis is supported by the finding that IL-12 increased only 1.9 fold, despite the increase in IFN-γ by 6.8-fold versus the control on day 14. Furthermore, we feel keenly the need to further study the possibility that regulatory T cells are involved in the inflammatory response elicited by semi-SWCNT exposure.

Finally, we evaluated the histopathology of and protein expression in lung tissue. Fibrotic change began decreasing from 14 days after semi-SWCNT exposure, but expression of TGF-β1 protein in lung tissue increased with time. The level of TGF-β1 in BAL fluid also was upregulated until day 28 after exposure. These results may be because immune cells containing semi-SWCNTs moved into BAL fluid [28]. In addition, expression of RANTES increased with time after semi-SWCNT exposure, unlike in our previous study in which we used mixed SWCNT [12]. RANTES has chemotactic functions in monocytes/macrophages, T cells, eosinophils and basophils [21]. Therefore, time-dependent increase in the amount of RANTES protein is associated with time-dependent recruitment of monocytes and eosinophils into the bloodstream from the bone marrow. Furthermore, MMPs are involved in the breakdown of the extracellular matrix during normal physiological processes such as embryonic development, reproduction, and tissue remodeling as well as disease processes such as arthritis and metastasis. MMP-9 is a major MMP and is known to be involved in lung fibrosis [29]. In this study, upregulation of MMP-9 protein peaked on day 14, and fibrotic lesions peaked from day 7 to day 14 after semi-SWCNT exposure.

These results suggest that the brain is the target organ of semi-SWCNTs brought into the lung, and conductance as well as length may be critical factors affecting the intensity and duration of the inflammatory response following SWCNT exposure.

## Supporting Information

Figure S1 **Comparison of body weights following exposure to single-walled carbon nanotubes (SWCNTs) and semi-SWCNTs.**
(DOC)

Figure S2 **Comparison of relative organ weight following exposure to SWCNTs and semi-SWCNTs.**
(DOC)

Table S1 **Comparison of hematological changes following exposure to single-walled carbon nanotubes (SWCNTs) and semi-SWCNTs. C: control group, T1: semi-SWCNT group, T2: SWCNT group.**
(DOC)
