# Supplementary material for: Biological Toxicity and Inflammatory Response of Semi-Single-Walled Carbon Nanotubes
Source: PLoS One. 2011 Oct 7;6(10):e25892. doi: 10.1371/journal.pone.0025892 (PMC3189226; doi:10.1371/journal.pone.0025892)

Figure S1. Comparison of body weights following exposure to single-walled carbon nanotubes (SWCNTs) and semi-SWCNTs.


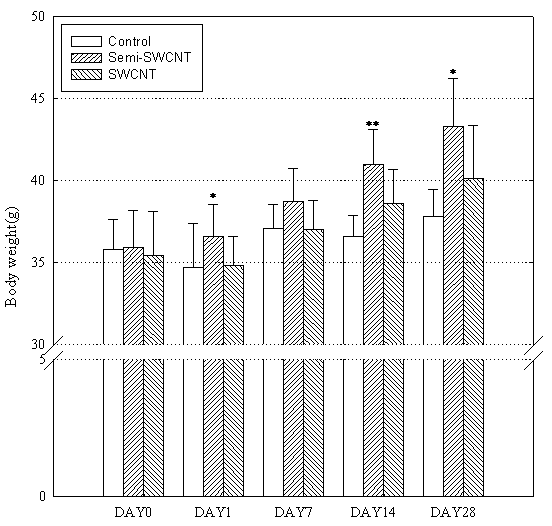

Supplement: Figure S1 — Comparison of body weights following exposure to single-walled carbon nanotubes (SWCNTs) and semi-SWCNTs. (DOC) [file pone.0025892.s001.doc]
