# Supplementary material for: Biological Toxicity and Inflammatory Response of Semi-Single-Walled Carbon Nanotubes
Source: PLoS One. 2011 Oct 7;6(10):e25892. doi: 10.1371/journal.pone.0025892 (PMC3189226; doi:10.1371/journal.pone.0025892)

Figure S2. Comparison of relative organ weight following exposure to SWCNTs and semi-SWCNTs.

(A) DAY 1


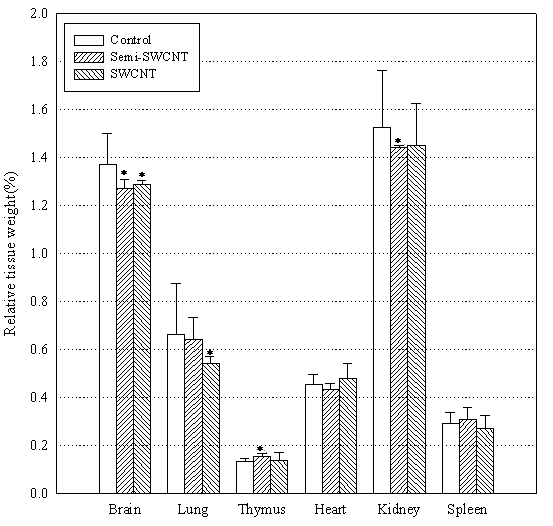


(B) DAY 7


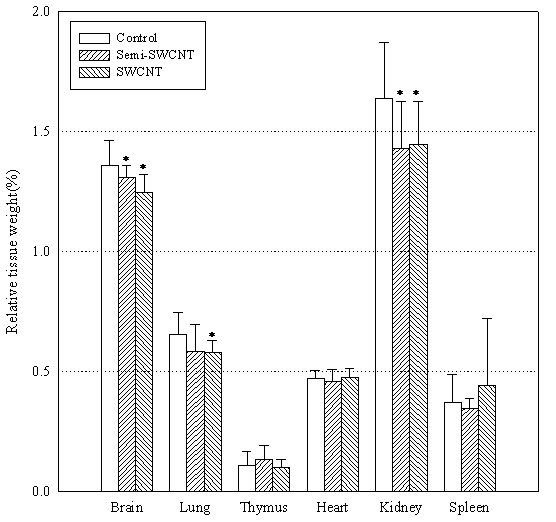


(C) DAY 14


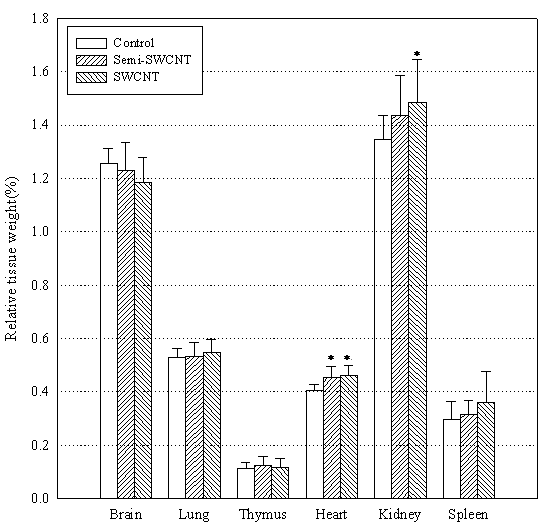


(D) DAY 28


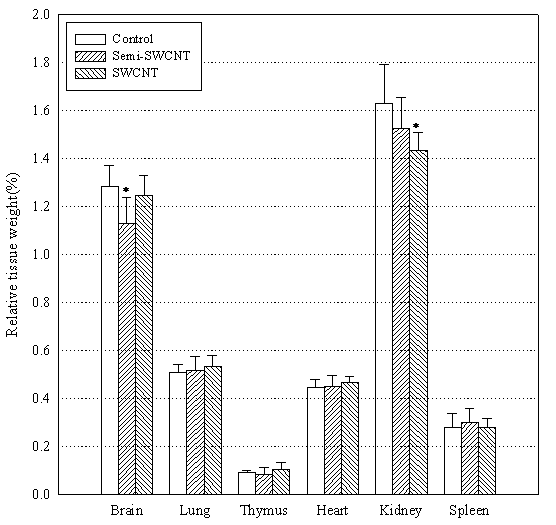

Supplement: Figure S2 — Comparison of relative organ weight following exposure to SWCNTs and semi-SWCNTs. (DOC) [file pone.0025892.s002.doc]
