# Supplementary material for: Biological Toxicity and Inflammatory Response of Semi-Single-Walled Carbon Nanotubes
Source: PLoS One. 2011 Oct 7;6(10):e25892. doi: 10.1371/journal.pone.0025892 (PMC3189226; doi:10.1371/journal.pone.0025892)
Supplement: Table S1 — Comparison of hematological changes following exposure to single-walled carbon nanotubes (SWCNTs) and semi-SWCNTs. C: control group, T1: semi-SWCNT group, T2: SWCNT group. (DOC) [file pone.0025892.s003.doc]

|  | | WBC | RBC | HGB | HCT | MCV | MCH | MCHC | PLT | RET | NEU | LYM | MON | EOS | BAS | LUC |
| --- | --- | --- | --- | --- | --- | --- | --- | --- | --- | --- | --- | --- | --- | --- | --- | --- |
|  |  | ×10^3^ µL | ×10^6^ µL | g/dL | % | fL | pg | g/dL | ×10^3^ µL | % | % | % | % | % | % | % |
| DAY  1 | C. | 2.6 ± 0.3 | 9.0 ± 0.3 | 13.4 ± 0.4 | 39.3 ± 0.3 | 49.5 ± 1.5 | 16.9 ± 1.0 | 34.2 ± 1.2 | 1423 ± 242 | 3.3 ± 0.5 | 21.9 ± 8.8 | 71.6 ± 8.3 | 1.2 ± 0.4 | 4.3 ± 0.2 | 0.4 ± 0.2 | 0.5 ± 0.3 |
|  | T1 | 2.8 ± 0.7 | 7.7 ± 0.4 | 12.8 ± 0.2 | 38.9 ± 1.3 | 50.3 ± 1.2 | 16.6 ± 0.8 | 33.0 ± 0.8 | 1391 ± 145 | 3.3 ± 0.3 | 19.8 ± 6.0 | 74.2 ± 6.5 | 1.9 ± 1.0 | 2.8 ± 0.7 | 0.2 ± 0.1 | 1.0 ± 0.6 |
|  | T2 | 3.4 ± 0.8 | 8.1 ± 0.4 | 14.0 ± 0.7 | 39.7 ± 1.7 | 49.3 ± 2.2 | 17.3 ± 1.2 | 35.2 ± 1.2 | 1367 ± 123 | 3.4 ± 0.4 | 26.3 ± 4.1 | 67.1 ± 4.5 | 1.5 ± 0.9 | 3.9 ± 1.0 | 0.4 ± 0.1 | 0.7 ± 0.2 |
| DAY  7 | C. | 4.9 ± 0.5 | 8.4 ± 0.3 | 13.4 ± 0.4 | 43.6 ± 2.3 | 52.0 ± 1.7 | 16.1 ± 0.1 | 30.7 ± 1.0 | 1518 ± 21 | 5.6 ± 1.3 | 57.4 ± 2.3 | 38.8 ± 2.8 | 1.0 ± 0.2 | 2.3 ± 0.6 | 0.1 ± 0.1 | 0.4 ± 0.4 |
|  | T1 | 4.6 ± 1.2 | 8.0 ± 0.3 | 12.8 ± 0.8 | 39.0 ± 1.5 | 48.7 ± 1.1 | 15.9 ± 0.8 | **32.7 ± 0.9*** | 1669 ± 187 | 5.0 ± 0.4 | **23.7 ± 4.7*** | **71.0 ± 4.0*** | 2.2 ± 0.4 | 2.2 ± 0.9 | 0.1 ± 0.1 | 0.8 ± 0.2 |
|  | T2 | 3.3 ± 0.7 | 8.7 ± 0.2 | 13.5 ± 0.6 | 41.4 ± 2.4 | **47.7 ± 2.1*** | 15.6 ± 0.4 | **32.6 ± 0.7*** | 1468 ± 83 | 4.4 ± 0.3 | **13.8 ± 3.6*** | **81.2 ± 5.0*** | 2.0 ± 0.7 | 2.4 ± 1.4 | 0.1 ± 0.0 | 0.5 ± 0.1 |
| DAY  14 | C. | 4.5 ± 1.2 | 8.3 ± 0.7 | 13.3 ± 1.4 | 40.6 ± 4.3 | 49.1 ± 1.5 | 16.1 ± 0.3 | 32.8 ± 1.0 | 1466 ± 259 | 4.3 ± 0.2 | 15.3 ± 7.3 | 80.2 ± 7.0 | 1.8 ± 0.2 | 2.0 ± 0.7 | 0.1 ± 0.1 | 0.6 ± 0.2 |
|  | T1 | 4.0 ± 1.1 | 8.9 ± 0.3 | 14.3 ± 0.3 | 45.2 ± 3.2 | 50.6 ± 2.4 | 16.1 ± 0.2 | 31.8 ± 1.5 | 1639 ± 203 | 4.4 ± 0.5 | 15.3 ± 1.4 | 80.1 ± 1.8 | **2.4 ± 0.3*** | 1.5 ± 0.4 | 0.1 ± 0.1 | 0.5 ± 0.2 |
|  | T2 | 6.6 ± 1.9 | 8.6 ± 0.1 | 13.5 ± 0.4 | 42.6 ± 0.6 | 49.6 ± 0.3 | 15.7 ± 0.4 | 31.7 ± 0.8 | 1643 ± 242 | 5.0 ± 0.5 | 19.0 ± 1.0 | 76.2 ± 1.2 | 2.2 ± 0.2 | 1.2 ± 0.3 | 0.2 ± 0.0 | **1.2 ± 0.3*** |
| DAY  28 | C. | 4.3 ± 0.8 | 8.8 ± 0.2 | 13.4 ± 0.5 | 41.5 ± 1.0 | 47.3 ± 0.8 | 15.2 ± 0.3 | 32.1 ± 0.4 | 1442 ± 30 | 3.1 ± 0.2 | 15.5 ± 7.1 | 80.8 ± 7.0 | 2.0 ± 0.7 | 1.1 ± 0.2 | 0.0 ± 0.1 | 0.5 ± 0.2 |
|  | T1 | **2.7 ± 0.2*** | 8.3 ± 0.5 | 13.1 ± 0.3 | 41.8 ± 0.3 | 50.4 ± 2.5 | 15.8 ± 0.8 | 31.4 ± 0.9 | 1455 ± 121 | 3.7 ± 0.2 | **46.0 ± 6.4*** | **46.1 ± 6.5*** | **4.35 ± 0.5*** | **2.8 ± 0.4*** | 0.2 ± 0.2 | 0.7 ± 0.3 |
|  | T2 | 3.0 ± 0.4 | 8.8 ± 0.8 | 13.5 ± 0.4 | 42.2 ± 1.5 | 48.1 ± 2.5 | 15.5 ± 0.9 | 32.1 ± 0.2 | 1571 ± 45 | 3.4 ± 0.5 | 14.2 ± 2.7 | 80.9 ± 2.9 | 2.7 ± 0.9 | 1.7 ± 0.7 | 0.1 ± 0.1 | 0.5 ± 0.4 |

Table S1. Comparison of hematological changes following exposure to single-walled carbon nanotubes (SWCNTs) and semi-SWCNTs. C: control group, T1: semi-SWCNT group, T2: SWCNT group.

Note: WBC: white blood cell; RBC: red blood cell; HGB: hemoglobin; HCT: hematocrit; MCV: mean corpuscular volume; MCH: mean corpuscular hemoglobin; MCHC: mean corpuscular hemoglobin concentration; PLT: platelets; RET: reticulocyte; NEU: neutrophils; LYM: lymphocytes; MON: monocytes; EOS: eosinophils; BAS: basophils; LUC: large unstained cells.
